# Supplementary material for: Insight into the Biological Activity of Hennosides—Glucosides Isolated from Lawsonia inermis (henna): Could They Be Regarded as Active Constituents Instead
Source: Plants (Basel). 2021 Jan 26;10(2):237. doi: 10.3390/plants10020237 (PMC7912310; doi:10.3390/plants10020237)
Supplement: Supplementary file 1 [file plants-10-00237-s001.pdf]

Table S1. Antioxidant activity of hennosides.

	Hennoside		Concentration of antioxidants (mM)			
			FRAP method		ABTS method	
	(μg/mL)	(mM)	NaCl	D'MEM/10%FCS	NaCl	D'MEM/FCS[#]
HA	0	0	0	0.41 ± 0.03	0	2.7 ± 0.1
	1	0.0028	0.03 ± 0.05	0.42 ± 0.05	0.01 ± 0.01	2.5 ± 0.5
	10	0.028	0.07 ± 0.09	0.41 ± 0.06	0.03 ± 0.01	2.6 ± 0.1
	100	0.28	0.08 ± 0.01	0.51 ± 0.05	0.06 ± 0.01	2.7 ± 0.1
	200	0.56	0.14 ± 0.01	0.58 ± 0.09	0.08 ± 0.01	2.6 ± 0.1
	500	1.4	0.46 ± 0.08	0.68 ± 0.03	0.15 ± 0.01	2.7 ± 0.2
	1000	2.8	0.92 ± 0.07	0.91 ± 0.03	0.32 ± 0.02	2.4 ± 0.5
HB	0	0	0	0.41 ± 0.03	0	2.7 ± 0.1
	1	0.0028	0.01 ± 0.01	0.43 ± 0.03	0.01 ± 0.01	2.7 ± 0.5
	10	0.028	0.14 ± 0.09	0.44 ± 0.03	0.01 ± 0.02	2.8 ± 0.2
	100	0.28	0.23 ± 0.14	0.56 ± 0.07	0.06 ± 0.01	3.0 ± 0.1
	200	0.56	0.28 ± 0.16	0.69 ± 0.16	0.11 ± 0.01	3.1 ± 0.2
	500	1.4	0.62 ± 0.28	0.73 ± 0.03	0.30 ± 0.01	2.8 ± 0.1
	1000	2.8	1.44 ± 0.07	0.96 ± 0.09	0.56 ± 0.02	2.6 ± 0.3
HC	0	0	0	0.41 ± 0.03	0	2.7 ± 0.1
	1	0.0028	0.03 ± 0.01	0.42 ± 0.01	0.01 ± 0.01	2.6 ± 0.1
	10	0.028	0.05 ± 0.01	0.44 ± 0.02	0.03 ± 0.01	2.9 ± 0.1
	100	0.28	0.09 ± 0.01	0.46 ± 0.01	0.01 ± 0.01	2.6 ± 0.3
	200	0.56	0.15 ± 0.01	0.55 ± 0.01	0.02 ± 0.01	2.7 ± 0.1
	500	1.4	0.35 ± 0.03	0.66 ± 0.04	0.09 ± 0.01	2.8 ± 0.1
	1000	2.8	0.68 ± 0.02	1.00 ± 0.03	0.21 ± 0.01	2.6 ± 0.5

HA – hennoside A; HB – hennoside B; HC – hennoside C;

NaCl – 0.9% NaCl; D'MEM-FCS D'MEM cell culture media supplemented with 10%FCS

([#]) Concentration of antioxidants in 10 times diluted D'MEM-FCS.

Table S2. Effect of hennosides on the erythrocyte lysis.

	Hennoside (μg/mL)	NaCl OD_{540}	NaCl/10%FCS OD_{540}	DMSO in NaCl dil. (x)	DMSO in NaCl OD_{540}
HA	0	0.049± 0.005	0.058 ± 0.004		
	1	0.051± 0.006	0.058 ± 0.010	500000	0.044 ± 0.003
	10	0.054 ± 0.006	0.058 ± 0.002	50000	0.044 ± 0.004
	100	0.055 ± 0.002	0.066 ± 0.003	5000	0.048 ± 0.005
	200	0.060 ± 0.002	0.074 ± 0.001	2500	0.048 ± 0.004
	500	0.080 ± 0.005	0.104 ± 0.008	1000	0.049 ± 0.005
	1000	0.103 ± 0.003	0.155± 0.011	500	0.056 ± 0.007
HB	0	0.049 ± 0.005	0.058± 0.004		
	1	0.047 ± 0.002	0.055 ± 0.004	500000	0.044 ± 0.003
	10	0.047 ± 0.004	0.058 ± 0.005	50000	0.044 ± 0.004
	100	0.058 ± 0.004	0.063 ± 0.001	5000	0.048 ± 0.005
	200	0.061± 0.003	0.073 ± 0.001	2500	0.048 ± 0.004
	500	0.077 ± 0.002	0.102 ± 0.001	1000	0.049 ± 0.005
	1000	0.098 ± 0.001	0.144 ± 0.003	500	0.056 ± 0.007
HC	0	0.049 ± 0.005	0.058 ± 0.004		
	1	0.053 ± 0.005	0.055 ± 0.002	500000	0.044 ± 0.003
	10	0.051 ± 0.006	0.057 ± 0.004	50000	0.044 ± 0.004
	100	0.053 ± 0.005	0.061 ± 0.001	5000	0.048 ± 0.005
	200	0.058 ± 0.003	0.063 ± 0.001	2500	0.048 ± 0.004
	500	0.059 ± 0.008	0.078 ± 0.001	1000	0.049 ± 0.005
	1000	0.070 ± 0.002	0.092 ± 0.003	500	0.056 ± 0.007

HA – hennoside A; HB – hennoside B; HC – hennoside C;

NaCl – hennosides diluted in 0.9% NaCl

NaCl-10%FCS – hennosides diluted in 0.9% NaCl supplemented with 10% FCS

DMSO in NaCl: Diluted DMSO used as a control; The results show no effect or only minor effect (500 x diluted) of DMSO on erythrocyte hemolysis.

Table S3. Effect of hennoside on methemoglobin formation

	Hennoside (μg/mL)	NaCl OD_{630}	NaCl/10%FCS OD_{630}
HA	0	0.096 ± 0.004	0.111 ± 0.004
	1	0.098 ± 0.003	0.112 ± 0.002
	10	0.097 ± 0.003	0.114 ± 0.003
	100	0.103 ± 0.001	0.115 ± 0.002
	200	0.108 ± 0.001	0.121 ± 0.004
	500	0.114 ± 0.002	0.134 ± 0.002
	1000	0.139 ± 0.003	0.149± 0.002
HB	0	0.096 ± 0.004	0.111 ± 0.004
	1	0.093 ± 0.004	0.113 ± 0.003
	10	0.100 ± 0.006	0.111 ± 0.008
	100	0.098 ± 0.008	0.116 ± 0.001
	200	0.100 ± 0.003	0.113 ± 0.003
	500	0.108 ± 0.006	0.116 ± 0.002
	1000	0.131 ± 0.004	0.124 ± 0.001
HC	0	0.096 ± 0.004	0.111 ± 0.004
	1	0.100 ± 0.002	0.110 ± 0.004
	10	0.097 ± 0.002	0.104 ± 0.006
	100	0.104 ± 0.003	0.107 ± 0.001
	200	0.106 ± 0.001	0.107 ± 0.003
	500	0.114 ± 0.002	0.116 ± 0.006
	1000	0.126 ± 0.001	0.122 ± 0.003

Methemoglobin formation studied based on the change in outdated hemoglobin OD_{630} values.

HA – hennoside A; HB – hennoside B; HC – hennoside C;

NaCl – hennosides diluted in 0.9% NaCl;

NaCl/10%FCS – hennosides diluted in 0.9% NaCl supplemented with 10% FCS.

Table S4. Effect of hennosides on concentration of antioxidants in cell-culture supernatants of human breast cancer cell lines and primary mesenchymal stem cells.

		Antioxidants concentration					
		24h		48h		72h	
	(μg/ml)	(mM)	(%)	(mM)	(%)	(mM)	(%)
		MDA 231					
HA	0	0.502 ± 0.025	100	0.627± 0.049	100	0.733 ± 0.064	100
	100	0.685	136	0.709	111	0.709	97
HB	0	0.502 ± 0.025	100	0.627± 0.046	100	0.733 ± 0.064	100
	100	0.698	139	0.792	126	0.899	123
HC	0	0.502 ± 0.025	100	0.627± 0.046	100	0.733 ± 0.064	100
	100	0.597	119	0.634	101	0.769	105
		MCF 7					
HA	0	0.560 ± 0.027	100	0.608 ± 0.031	100	0.688 ± 0.032	100
	100	0.651	116	0.725	119	0.807	105
HB	0	0.560 ± 0.027	100	0.608 ± 0.031	100	0.688 ± 0.032	100
	100	0.636	114	0.717	118	0.751	104
HC	0	0.560 ± 0.027	100	0.608 ± 0.031	100	0.688 ± 0.032	100
	100	0.698	125	0.679	112	0.697	99
		PDL-MSCs					
HA	0	0.522 ± 0.037	100	0.549 ± 0.093	100	0.679 ± 0.069	100
	100	0.615	118	0.775	141	0.914	135
HB	0	0.522 ± 0.037	100	0.549 ± 0.093	100	0.679 ± 0.069	100
	100	0.612	117	0.698	127	0.780	112
HC	0	0.522 ± 0.037	100	0.549 ± 0.093	100	0.679 ± 0.069	100
	100	0.584	112	0.592	108	0.601	86
		PB-MSCs					
HA	0	0.436 ± 0.014	100	0.462 ± 0.041	100	0.592 ± 0.029	100
	100	0.563	129	0.611	132	0.707	119
HB	0	0.436 ± 0.014	100	0.462 ± 0.041	100	0.592 ± 0.029	100
	100	0.616	141	0.635	137	0.644	109
HC	0	0.436 ± 0.014	100	0.462 ± 0.041	100	0.592 ± 0.029	100
	100	0.503	117	0.541	117	0.590	100

HA – hennoside A; HB – hennoside B; HC – hennoside C.
MDA 231 and MCF-7 – human breast cancer cell lines; PDL-MSC - primary human periodontal ligament; PB-MCS - primary human peripheral blood mesenchymal stem cells.
The concentration of antioxidants in the cell culture supernatant was determined by FRAP assay.
The concentration of antioxidants without hennosides is mean ± SD of three independent measurements; The concentration of antioxidants with hennosides are mean values of one measurement performed in triplicates.
